# All Known Human Rhinovirus Species Are Present in Sputum Specimens of Military Recruits During Respiratory Infection

**DOI:** 10.3390/v1031178

**Published:** 2009-12-04

**Authors:** Carita Savolainen-Kopra, Soile Blomqvist, Svetlana Kaijalainen, Ulla Jounio, Raija Juvonen, Ari Peitso, Annika Saukkoriipi, Olli Vainio, Tapani Hovi, Merja Roivainen

**Affiliations:** 1 Gastrointestinal Infections Unit, Department of Infectious Disease Surveillance and Control, National Institute for Health and Welfare (THL), P.O. Box 30, FI-00271 Helsinki, Finland; E-Mails: soile.blomqvist@thl.fi (S.B.); svetlana.kaijalainen@thl.fi (S.K.); tapani.hovi@thl.fi (T.H.); merja.roivainen@thl.fi (M.R.); 2 Department of Medical Microbiology, Institute of Diagnostics, University of Oulu, Oulu, Finland; E-Mails: ujounio@paju.oulu.fi (U.J.); olli.vainio@oulu.fi (O.V.); 3 Clinical Microbiology Laboratory, Oulu University Hospital, Oulu, Finland; 4 Department of Otorhinolaryngology, Kainuu Central Hospital, Kajaani, Finland; E-Mail: raija.juvonen@kainuu.fi (R.J.); 5 Centre for Military Medicine, Finnish Defence Forces, Lahti, Finland; E-Mail: ari.peitso@mil.fi (A.P.); 6 Child and Adolescent Health and Wellbeing Unit, Lifecourse and Services Department, National Institute for Health and Welfare (THL), Oulu, Finland; E-Mail: annika.saukkoriipi@thl.fi (A.S.)

**Keywords:** human rhinovirus, genetic distribution, adult, asthma

## Abstract

Human rhinoviruses (HRV) are known to cause common cold as well as more complicated respiratory infections. HRV species -A, -B and -C have all been associated with lower respiratory infections and exacerbations of asthma. However, the type distribution of strains connected to different kinds of lower respiratory conditions is not clearly known. We have analysed the presence of HRV in sputum specimens derived from military recruits with and without pre-diagnosed asthma at times of acute respiratory infection (CIAS Study, 2004–2005). The analysis was performed with HRV and HEV real-time RT-PCR assays. Subsequently we studied type distribution of HRV strains by genetic typing in the VP4/VP2 genomic region. In total 146 (38.8%) specimens were HRV-positive and 36 (9.3%) HEV-positive. No difference was found in HRV detection between the asthmatic *vs*. non-asthmatic patients. Most of the genetically typed strains, 18 (62.1%), belonged to HRV-A, while HRV-B strains constituted five (17.2%) of the HRV-positive strains. HRV-C strain was typed four times from the HRV-positive cases and a HEV-D strain twice. We further typed six HEV positive strains in the partial VP1 region. Three of these belonged to HRV-A and three to HEV-D. HRV-A strains were discovered throughout the study period, while HRV-C strains originated from winter and spring specimens. Interestingly, four out of five typed HRV-B strains originated from the summer season specimens.

## 1. Introduction

Human rhinoviruses (HRVs) are known to cause common cold as well as more complicated respiratory infections. Association of HRV with lower respiratory tract infections is well documented: HRV RNA has been found in lower respiratory tract cells [1], the bronchial epithelial cells are susceptible to HRV infection [2], HRVs cause cytopathic effect and induce production of interleukin (IL)-6, IL-8, IL-16, granulocyte macrophage colony-stimulating factor, and RANTES (regulated on activation, normal T expressed and secreted) [3,4], and HRV RNA has been detected in tissues of lower airways by *in situ* hybridization [4]. HRVs infect the airway epithelium, generate local and systemic immune responses, as well as neural responses, which induce inflammation and airway hyper responsiveness (reviewed by Papadopoulos *et al*. [5]). Clinical evidence suggests association of HRVs to various lower respiratory diseases: bronchiolitis, chronic obstructive pulmonary disease, pneumonia, and asthma exacerbations (reviewed by Hayden [6]). HRVs belong, together with human enteroviruses (HEV), to genus *Enterovirus* in the family *Picornaviridae* and are currently divided in three species: *HRV-A* and *HRV-B* [7] and the recently discovered, proposed new species, *HRV-C* [8]. All species have been associated to lower respiratory infections and exacerbations of asthma [9–13]. However, the type distribution of strains connected to different kinds of lower respiratory conditions is not clearly known. In addition to HRVs, HEVs are also known to be present in respiratory samples. HEVs are currently divided in the species HEV-A, HEV-B, HEV-C, and HEV-D [14] . This study analysed respiratory samples from adult military conscripts with and without previously diagnosed asthma. We aimed at elucidating the role of HRVs in respiratory infections of adults. Furthermore, we aimed to study genetic variation of HRVs in the Finnish adult population, as previous studies concentrated mainly on children.

## 2. Results and Discussion

### 2.1. Real-time RT-PCR

We analyzed 386 sputum specimens derived from military recruits with or without pre-diagnosed asthma. The specimens were taken during 2004–2005 at times of clinically diagnosed respiratory infections. Out of 386 specimens 146 (37.8%) were tested positive in HRV real-time RT-PCR. All specimens were also studied with human enterovirus (HEV) real-time RT-PCR. A total of 36 specimens (9.3%) were positive with the assay for HEV. All HEV-positive specimens were also positive with HRV assay with one exception. The results did not differ between the asthmatics and non-asthmatics (data not shown). The connection of HRV- and HEV-positivity to other aspects, such as severity of the symptoms, will be studied and published elsewhere.

According to the results of HRV real-time RT-PCR almost 40% of the specimens were HRV-positive. This is in accordance with previous findings of HRV detection rate in adults in other geographical regions [15,16]. It has also been shown earlier that HRV infection of lower airway tissues is a frequent finding during a cold [17]. HRVs are also abundantly found in adult population during respiratory infections. This fact is possibly emphasized in a semi-closed environment of a garrison, where the infection pressure is great, and infections are easily spread. Other respiratory pathogens of the specimens were tested and will be reported elsewhere.

### 2.2. Genetic typing in the capsid coding region

HRV-positive specimens were subjected to genetic typing in the VP4/VP2 genomic region according to a previously described method [18]. From 146 HRV-positive specimens, 55 gave a positive result in the RT-PCR for genetic typing, while 91 remained negative. Reliable sequence typing results were obtained for 29 strains (Table 1). Most of the genetically typed strains (62.1%) belonged to HRV-A. Six different genetic types were discovered (Table 1). HRV-B strains accounted for 17.2% of the typed strains. Three distinct genetic types were discovered (Table 1). Four HRV-C strains (14.3%) were genetically typed. Two strains belonging to HEV-D were also typed among the HRV-real-time RT-PCR-positive strains (Table 1). Both were also positive in the HEV real-time RT-PCR (Table 1).

Nine HEV-positive specimens were selected for genetic typing in the partial VP1 coding region [19], including those with the lowest C_t_ values (data not shown) as well as the one specimen which was not positive for HRV, but positive in the HEV assay. All studied strains gave a positive result in the semi-nested RT-PCR, however, only six of them were successfully sequenced. Three of these were HEV-D strains, while the other three were genetically closest to HRV51 (Table 1).

### 2.3. Epidemiological characteristics

**Figure 1.**
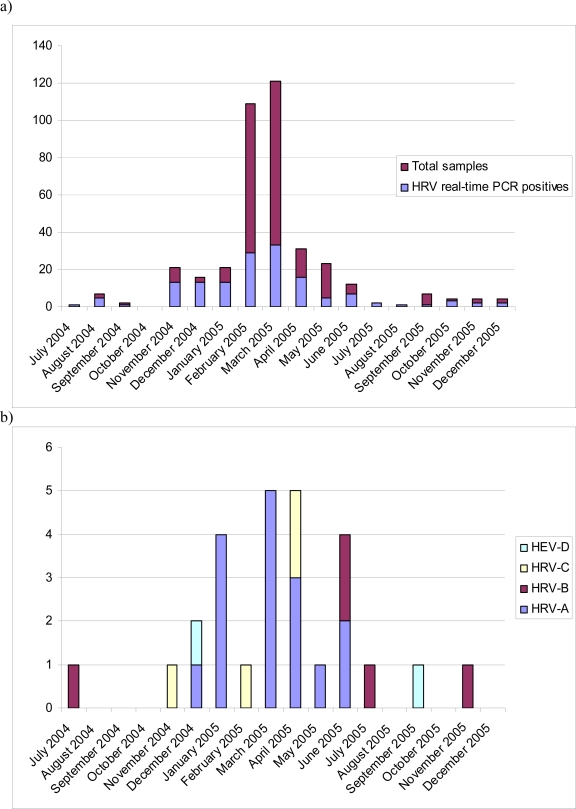
a) Monthly distribution of HRV findings. b) Monthly distribution of different species.

HRVs were detected abundantly whenever sampled (Figure 1a). In the results of genetic typing, HRV-A strains prevailed outnumbering all other discovered species. Recent studies have provided similar results [15]. HRV-A strains appear to be a frequent finding in all age groups in both upper [18,20] and lower [15,21] respiratory infections. HRV-A strains HRV16 and HRV51 were detected several times during January 2005 and March–April 2005, respectively, suggesting local outbreaks (Table 1). HRV-A strains were also discovered steadily throughout the year, which can be explained by their ubiquitous character (Figure 1b). HRV-B strains, on the other hand, were discovered scarcely as in other recent reports. Interestingly four out of five strains were collected during summer months (Figure 1b).

Although the number of strains is too small to draw definite conclusions, it is tempting to speculate whether this has to do with their infrequent discovery in recent studies. It is possible that HRV-B strains are missed if they preferably circulate during summer months, while the collection of respiratory samples is weighted on winter months. We have previously shown an even distribution of HRV-A and HRV-B strains in upper respiratory infections derived from young children [20]. In that study the samples were cultured in cell lines, which possibly favoured HRV-B strains. Nevertheless, sampling also took place throughout the year, thus enabling sampling of any summer-HRV-B strains as well. The hypothesis of a season-restricted epidemiology of HRV-B will require further studying to cover several independent strains. In addition to characteristics of HRV-B in this study, it is noteworthy that one of the summer-HRV-B strains in this study (K1206_290704) has close genetic field relatives reported only in China (GenBank BLAST search, 5 August 2009). This finding may reflect the fact that strains possibly causing mild, self limited illness do not often get caught in typing efforts when sample collection is limited to hospitalized patients. This, in turn, shows that the biodiversity of circulating HRV strains by far outnumbers the commonly typed ones.

HRV-C strains were discovered in very limited numbers in this study. The genetic distances between the strains were large (1.2–27.7%). This has been reported in other recent studies as well [15,22–24]. This finding supports the assumption that there will be many sequence spaces to be filled once more HRV-C strains are characterized. The number of HRV-C findings is too small to suggest conclusions about their association to lower respiratory infection in adults. Furthermore, as mentioned above, we did not analyse the severity of respiratory symptoms of military recruits in this study. It is possible that HRV-species differ in the severity of infections among asthmatics.

A HEV-D strain, genetically most closely related to HEV68/HRV87 [25,26], was typed twice in the VP4/VP2 coding region and three times in the partial VP1 coding region. These are among the first reported findings of HEV68 in clinical samples in Finland, although the seroprevalence of HEV68 antibodies in Finland exceeds 98% (Roivainen, M., Klemola, P., Kaijalainen, S., unpublished result). HEV68 has rarely been discovered in studies conducted in other countries. The high seroprevalence suggests a wide circulation of HEV68, but it is likely that the virus strains do not end up being typed because of the mild symptoms. No other HEV strains were discovered among the sequenced strains.

### 2.4. Limitations of the study and of the methods in the diagnostics of HRV and HEV

The specimens of this study were sputa collected at times of any respiratory infection. Thus the sample collection does not provide a direct link to lower respiratory disease, as specimens were also taken during upper respiratory infections. It also has to be mentioned here that we cannot rule out the fact that the specimens may have been contaminated with pharyngeal HRV. The sputum in the specimens, however, at least partly originated from bronchioles. Thus the results may refer to the abundance of HRV RNA in the lower respiratory tract.

The number of HRV-positive specimens in real-time RT-PCR, but negative in RT-PCR for genetic typing was high (91/146). Furthermore reliable sequence was only received from 29/55 of the HRV sequencing RT-PCR positive specimens. Because of the small proportion of the genetically typed strains, there may exist a bias in the distribution of the typed strains *vs*. the real distribution. The high sensitivity of real-time methods partly explains negative typing results of specimens with high C_t_-values. The method used for genetic typing is not sensitive enough to enable typing of all real-time RT-PCR-positive HRV strains. The number of HRV genomes present may just be too low. These small numbers of genomes may also be remnants of a previous infection, not necessarily related to the current symptoms. Moreover, primer mismatch may explain negative results of the genetic typing method in cases when C_t_-values have been low. Possibly the amplification of the RT-PCR for genetic typing has been ineffective due to primer mismatch causing low amount of the PCR product. One factor explaining the results may also be cross-reaction of the presumably HRV-specific real-time RT-PCR with HEV strains. In this study we did not aim to evaluate the assays, but to present observations of their use. Even though only HEV-D strains were discovered in this study, other HEV strains are known to be present during respiratory infections. They may have high sequence homology with HRV in the 5′non-coding region where the primer and probe targets in real-time RT-PCR lie. The primers used in our genetic typing method for HRV do not recognize all species of HEV and even the semi-nested partial VP1 typing assay [19] failed to disclose the genetic type of the weaker strains. It is possible that some “HRV-positives” are actually HEV positives. It is also possible that some HRV strains are recognized by the presumably HEV-specific real-time RT-PCR. These are facts that are currently impossible to resolve even with methods of cell culture, as some serotypes of enteroviruses and HRV-C strains do not multiply in conventional laboratory cell lines.

## 3. Experimental Section

### 3.1. Samples

This work is part of a larger CIAS (Cold, Infections and Asthma) study about risk factors for asthma and respiratory infections in Finnish military conscripts [27]. Briefly, 892 men from a total of 3,697 men making up two intakes of conscripts to the Kainuu Brigade in Kajaani, Northern Finland, in July 2004 and January 2005, were enrolled in the CIAS study. These included all 224 men with a diagnosis of asthma in previous health examinations or the call-up examination, and 668 randomly chosen men without asthma. Their ages ranged from 17.4 to 29.6 years (median 19.6). The enrollment procedure has been described in detail previously [27]. Each man was followed to the end of his service time lasting 6 to 12 months, according to military duties. The protocol was accepted by the Medical Ethics Committee of Kainuu Central Hospital, Kajaani, Finland, and all the participants signed a declaration of informed consent.

A sputum sample was taken in the acute phases of each episode of infection, diagnosed on the basis of symptoms and clinical findings as described in detail previously [27]. The military conscripts having at least one infection episode during military service were included in this analysis. The specimen collection consisted of 386 sputum samples. The specimens were stored at −70°C prior to virological analysis. The viral RNA was isolated from 100 μL of sample with RNAeasy Mini Kit (Qiagen Gmbh, Hilden, Germany) using QIAcube automatic nucleic acid extractor.

### 3.2. Real-time RT-PCR

Real-time RT-PCR for detection of HRV was performed as described in [28]. The cut-off threshold was set at approximately 8% of the strongest positive control (HRV2 dilution of 10^−3^) and all plots rising above it were considered positive. Real-time RT-PCR for detection of HEV was performed according to the method by Centers for Disease Control and Prevention, Atlanta, USA (Nix, W.A.; Kilpatrick, D.R.; personal communication). The cut-off threshold was set at 10% of the strongest positive control (Coxsackievirus B5 dilution of 10^−2^).

### 3.3. RT-PCR and sequencing

RT-PCR targeting the VP4/VP2 coding region of all samples positive for HRV in real-time RT-PCR was performed as described in [18]. In cDNA synthesis a mixture of three primers was used. In addition to 9565 [18,20] primer 91575-1: 5′- TCD GGN ADY TTC CAV CAC CAN CC -3′ and 91575-2: 5′- TCD GGX ADY TTC CAV CAC CAX CC -3′ were used (D= A, G, T; Y= C, T; V= A, C, G; N= A, C, G, T; X= inosine). The location of the target sequence of these primers is identical to that of 9565. RT-PCR targeting partial 5′-VP1 was performed for nine samples positive for HEV in real-time RT-PCR as described in [19]. Sequencing and raw data analysis was executed as described in [18]. Multiple sequence alignments were made with ClustalX 1.83 [29]. Phylogenetic analysis was performed utilizing MEGA4 software [30] using Maximum composite likelihood model [31] with 1000 bootstrap replicates [32]. The sequences generated in this work have been submitted to GenBank under accession numbers GQ466456–GQ466490.

## 4. Conclusions

In conclusion, this study shows that the genetic type distribution of HRV strains in sputum samples of adult military recruits mirrors the distribution of overall HRV diversity. All known species are represented, with HRV-A strongly prevailing. While no significant differences were found between asthmatic *vs*. non-asthmatic patients, several suggestions were made to elucidate patterns of HRV epidemiology.

## Figures and Tables

**Figure 2.**
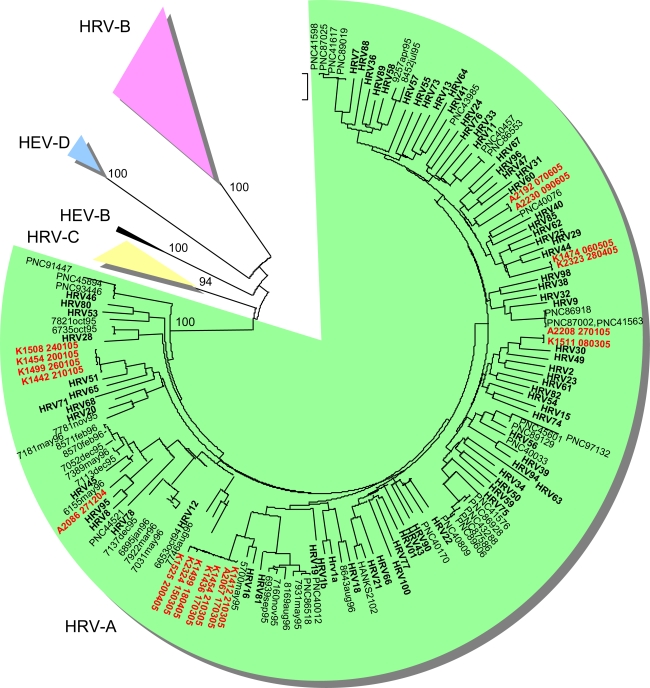
Phylogenetic tree depicting genetic relationships of HRV-A strains (green background) in Finland in the VP4/VP2 coding region. Strains of this study are shown in red and prototype strains in bold. Finnish strains from [20] are coded with xxxx_month_year and strains from [18] coded with PNCyyyy.

**Figure 3.**
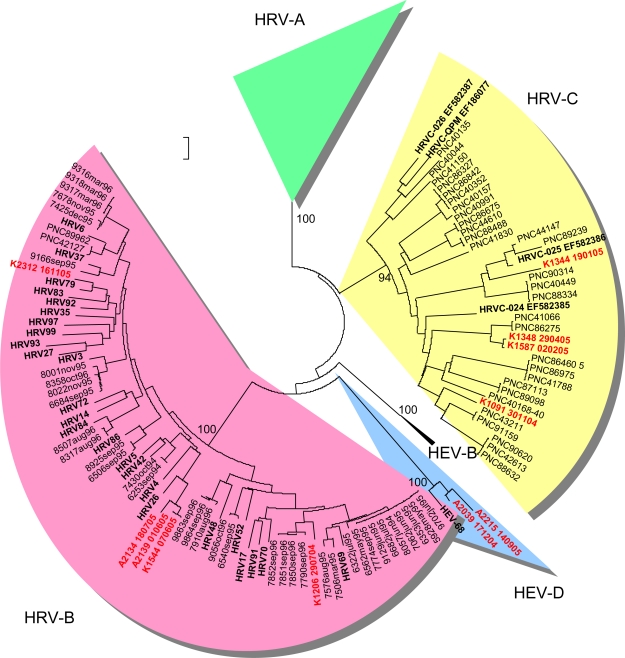
Phylogenetic tree depicting genetic relationships of HRV-B (pink background) and HRV-C (yellow background) strains in Finland in the VP4/VP2 coding region. For more details refer to legend for Figure 1.

**Table 1.** Nucleotide similarities of the genetically typed strains to prototype strains.

			Typing in the VP4/VP2 coding region		Typing in the partial VP1 coding region	

Collection date	Strain ID	HRV/HEV Real time RT-PCR result	The closest prototype strain or GenBank match*	Genetic similarity	The closest prototype strain or GenBank match	Genetic similarity
7/29/2004	1206	HRV+/HEV−	HRV91 (HRV-B)	90.0 %		
11/30/2004	1091	HRV+/HEV+	NAT045 (HRV-C)	82.5%	untypable^	
12/9/2004	2119	HRV+/HEV+	untypable^		HEV68 (HEV-D)	86.3%
12/17/2004	2039	HRV+/HEV+	HRV87 (HEV-D)	91.8%	untypable^	
12/17/2004	1105	HRV+/HEV+	untypable^		HEV68 (HEV-D)	86.3%
12/27/2004	2086	HRV+/HEV−	HRV95 (HRV-A)	92.5%		
1/20/2005	1454	HRV+/HEV−	HRV51 (HRV-A)	92.5%		
1/21/2005	1442	HRV+/HEV+	HRV51 (HRV-A)	92.3%	HRV51 (HRV-A)	88.4%
1/24/2005	1508	HRV+/HEV+	HRV51 (HRV-A)	93.4%	HRV51 (HRV-A)	88.4%
1/26/2005	1499	HRV+/HEV+	HRV51 (HRV-A)	93.1%	HRV51 (HRV-A)	88.4%
1/27/2005	2208	HRV+/HEV−	HRV30 (HRV-A)	93.8%		
2/2/2005	1587	HRV+/HEV+	06-339 (HRV-C)	99.0 %		
2/22/2005	2145	HRV−/HEV+	untypable^		HEV68 (HEV-D)	86.0%
3/8/2005	1511	HRV+/HEV−	HRV30 (HRV-A)	94.0 %		
3/15/2005	2324	HRV+/HEV−	HRV16 (HRV-A)	92.9%		
3/17/2005	2067	HRV+/HEV−	HRV16 (HRV-A)	92.7%		
3/17/2005	1436	HRV+/HEV−	HRV16 (HRV-A)	92.9%		
3/21/2005	1412	HRV+/HEV−	HRV16 (HRV-A)	92.6%		
3/21/2005	1454	HRV+/HEV+	HRV16 (HRV-A)	92.7%		
4/18/2005	1344	HRV+/HEV−	N38 (HRV-C)	87.3%		
4/18/2005	1499	HRV+/HEV−	HRV16 (HRV-A)	94.6%		
4/20/2005	1525	HRV+/HEV−	HRV16 (HRV-A)	91.5%		
4/28/2005	2323	HRV+/HEV−	HRV29 (HRV-A)	91.6%		
4/29/2005	1348	HRV+/HEV+	N27 (HRV-C)	98.2%		
5/6/2005	1474	HRV+/HEV−	HRV44 (HRV-A)	91.3%		
6/1/2005	2139	HRV+/HEV−	HRV26 (HRV-B)	92.8%		
6/7/2005	2192	HRV+/HEV+	HRV55 (HRV-A)	85.0 %		
6/7/2005	1544	HRV+/HEV−	HRV26 (HRV-B)	92.9%		
6/9/2005	2230	HRV+/HEV−	HRV57 (HRV-A)	89.0 %		
7/18/2005	2134	HRV+/HEV−	HRV26 (HRV-B)	89.5%		
9/14/2005	2215	HRV+/HEV+	HEV 68 (HEV-D)	87.6%	untypable^	
11/16/2005	2312	HRV+/HEV−	HRV79 (HRV-B)	92.0 %		

* **The closest GenBank match used for HRV-C, prototype strains have not been designated to HRV-C**

^ **Untypable: unreliable sequence with low, overlapping or ambiguous peaks**
